# Crystal structure and Hirshfeld surface analysis of (*E*)-2,4-di-*tert*-butyl-6-[(3-chloro-4-methyl­phenyl­imino)­meth­yl]phenol

**DOI:** 10.1107/S2056989018016377

**Published:** 2018-11-30

**Authors:** Sevgi Kansiz, Mustafa Macit, Necmi Dege, Vadim A. Pavlenko

**Affiliations:** a Ondokuz Mayıs University, Faculty of Arts and Sciences, Department of Physics, 55139, Kurupelit, Samsun, Turkey; b Department of Chemistry, Faculty of Arts and Sciences, Ondokuz Mayıs, University, 55139, Samsun, Turkey; c Taras Shevchenko National University of Kyiv, Department of Chemistry, Volodymyrska Str., 64, 01601 Kiev, Ukraine

**Keywords:** crystal structure, Schiff bases, 3-chloro-4-methyl­phenyl­imino, Hirshfeld surface

## Abstract

The title Schiff base compound, C_22_H_28_ClNO, shows mirror symmetry with all its non-H atoms, except the *tert*-butyl groups, located on the mirror plane. There is an intra­molecular O—H⋯N hydrogen bond present forming an *S*(6) ring motif. In the crystal, the mol­ecules are connected by C—H⋯π inter­actions, generating a three-dimensional supra­molecular structure. Hirshfeld surface analysis and two dimensional fingerprint plots were used to analyse the inter­molecular inter­actions present in the crystal, indicating that the most important contributions for the crystal packing are from H⋯H (68.9%) and C⋯H/H⋯C (11.7%) inter­actions.

## Abstract 2

The mol­ecule has mirror symmetry with the all non-H atoms, except *tert*-butyl groups, located on the mirror plane. An intra­molecular O—H⋯N hydrogen bond forms an *S*(6) ring motif. In the crystal, the mol­ecules are connected by C—H⋯π inter­actions

## Chemical context   

In coordination chemistry, Schiff bases have found wide use as ligands (Calligaris *et al.*, 1972 ▸; Hökelek *et al.*, 2004 ▸; Moroz *et al.*, 2012 ▸; Kansiz *et al.*, 2018 ▸). Schiff bases are important for various areas of chemistry and biochemistry because of their biological activity (El-masry *et al.*, 2000 ▸) and photochromic properties and have applications in various fields such as the measurement and control of radiation intensities in imaging systems and optical computers (Elmalı *et al.*, 1999 ▸), electronics, optoelectronics and photonics (Iwan *et al.*, 2007 ▸). They have been used as starting materials in the synthesis of many important medicinal substances. In the present study, a new Schiff base compound was synthesized and its crystal structure determined by X-ray diffraction. In addition, to understand the inter­molecular inter­actions in the crystal structure, Hirshfeld surface analysis was performed.
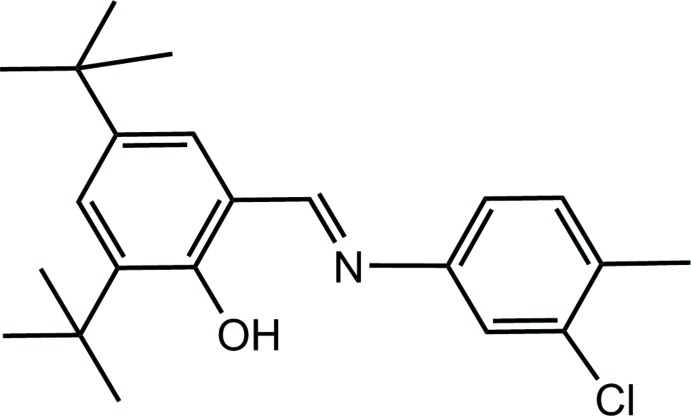


## Structural commentary   

**Figure 1:**
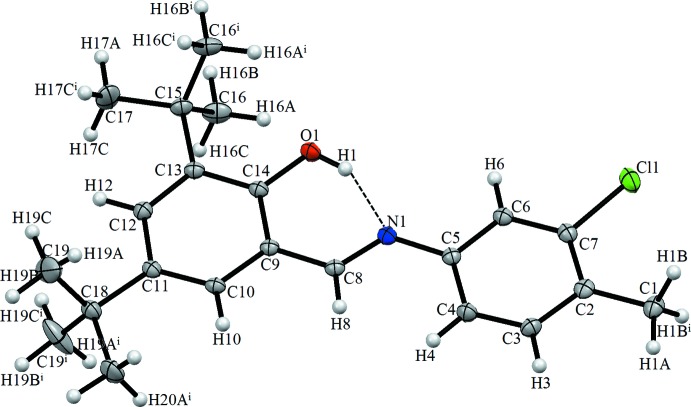
The mol­ecular structure of the title compound, showing the atom labelling. Displacement ellipsoids are drawn at the 10% probability level. Symmetry code: (i) *x*, −*y* + 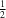, *z*.

The mol­ecular structure of the title compound is illustrated in Fig. 1 ▸. The title Schiff base compound shows mirror symmetry with all the non-H atoms, except the *tert*-butyl groups, located on the mirror plane. The C14—O1 bond distance is 1.349 (3) Å, the C8=N1 and C5—N1 bond lengths are 1.278 (4) and 1.412 (4) Å, respectively, and the C7—Cl1 bond distance is 1.744 (3) Å. There is an intra­molecular O—H⋯N hydrogen bond present (Table 1 ▸), forming an S(6) ring motif.

## Supra­molecular features   

**Figure 2:**
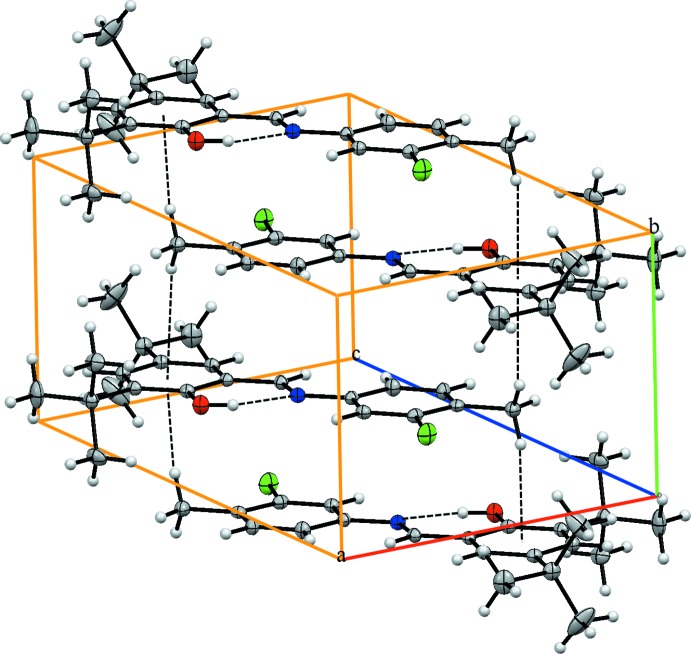
A view of the crystal packing of the title compound. Dashed lines denote the intra­molecular and inter­molecular hydrogen bonds.

In the crystal, the mol­ecules are connected by C1—H1*B*⋯π inter­actions, generating a three-dimensional supra­molecular structure (Table 1 ▸ and Fig. 2 ▸).

## Database survey   

There are no previous reports of the title structure. However, several related structure have been reported (CSD, version 5.39, update May 2018; Groom *et al.*, 2016 ▸), including bis­{(*E*)-1-[(3-chloro-4-methyl­phenyl­imino)­meth­yl]naphthalen-2-olate-*N*,*O*}copper(II) (SICXOU; Toprak *et al.*, 2018 ▸), 2-{(*E*)-[(3-chloro-4-methyl­phen­yl)imino]­meth­yl}-4-(tri­fluoro­meth­oxy)phenol (TERTUI; Atalay *et al.*, 2017 ▸), {2,2′-[4-chloro-5-methyl-*o*-phenyl­enebis(nitrilo­methyl­idyne)]dipheno­lato}nick­el(II) (WABDEK; Wang, 2010 ▸) and 4-[(*E*)-(3-chloro-4-methyl­phen­yl)imino­meth­yl]-2-meth­oxy-3-nitro­phenyl acetate (GAPPOE; Su *et al.*, 2012 ▸). In all four compounds, the C—Cl bond lengths vary from 1.724 to 1.743 Å. In the title compound, the C7—Cl1 bond length is 1.744 (3) Å.

## Hirshfeld surface analysis   

**Figure 3:**
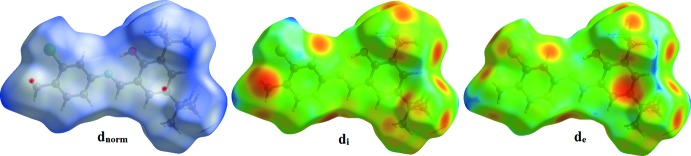
The Hirshfeld surface of the title compound mapped over *d*_norm_, *d*_i_ and *d*_e_.

The Hirshfeld surface analysis was performed using *CrystalExplorer* (Turner *et al.*, 2017 ▸). The Hirshfeld surfaces and their associated two-dimensional fingerprint plots were used to qu­antify the various inter­molecular inter­actions in the synthesized complex. The Hirshfeld surfaces mapped over *d*_norm_, *d*_e_ and *d*_i_ are illustrated in Figs. 3 ▸ and 4 ▸. The red spots on the surfaces indicate the inter­molecular contacts involved in strong hydrogen bonds and inter­atomic contacts (Şen *et al.*, 2017 ▸; Kansız & Dege, 2018 ▸; Sen *et al.*, 2018 ▸; Gumus *et al.*, 2018 ▸). The Hirshfeld surfaces were calculated using a standard (high) surface resolution with the three-dimensional *d*_norm_ surfaces mapped over a fixed colour scale of −0.031 (red) to 2.139 (blue) a.u. The red spots identified in Fig. 3 ▸ correspond to the near-type H⋯π contacts resulting from the C—H⋯π inter­actions (Table 1 ▸).

**Figure 5:**
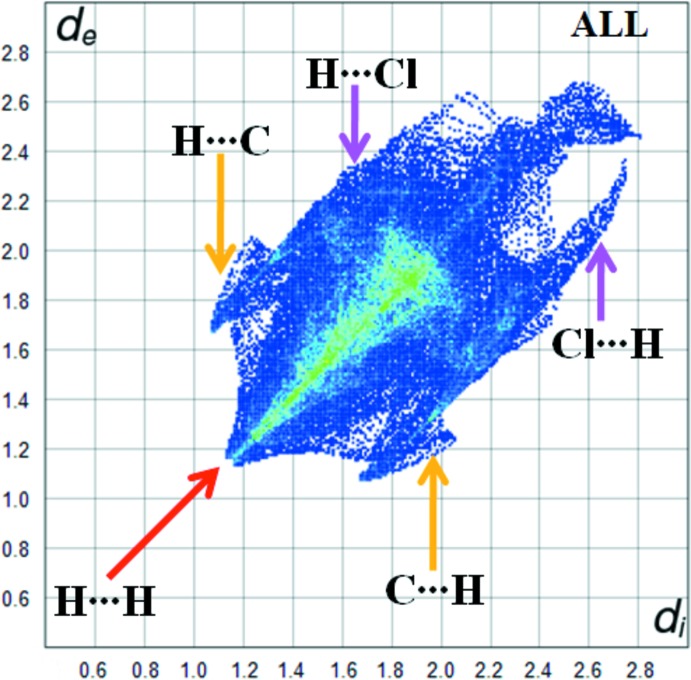
Overall fingerprint plot for the title compound.

**Figure 6:**
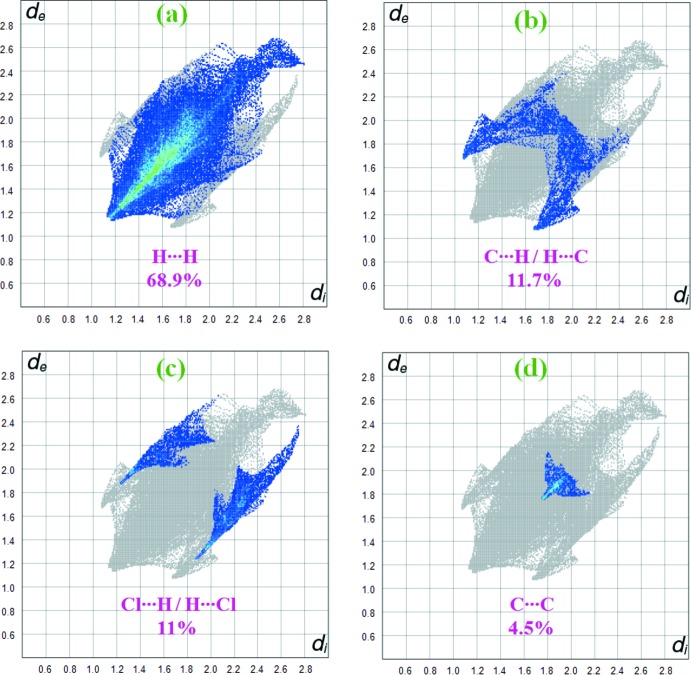
Two-dimensional fingerprint plots with a *d*_norm_ view of the (*a*) H⋯H (68.9%), (*b*) C⋯H/H⋯C (11.7%), (*c*) Cl⋯H/H⋯Cl (11%) and (*d*) C⋯C (4.5%) contacts in the title compound.

Fig. 5 ▸ shows the two-dimensional fingerprint of the sum of the contacts contributing to the Hirshfeld surface represented in normal mode. The graph shown in Fig. 6 ▸(*a*) (H⋯H) shows the two-dimensional fingerprint of the (*d*_i_, *d*_e_) points associated with hydrogen atoms. It is characterized by an end point that points to the origin and corresponds to *d*_i_ = *d*_e_ = 1.08 Å, which indicates the presence of the H⋯H contacts in this study (68.9%). The graph shown in Fig. 6 ▸(*b*) shows the (C⋯H/H⋯C) contacts between the carbon atoms inside the surface and the hydrogen atoms outside the Hirshfeld surface and *vice versa*, which contribute 11.7%. There are two symmetrical wings on the left and right sides. Furthermore, there are also Cl⋯H/H⋯Cl (11%), C⋯C (4.5%), C⋯N/N⋯C (2.2%), O⋯H/H⋯O (1.3%) and N⋯H/H⋯N (0.4%) contacts.

**Figure 7:**
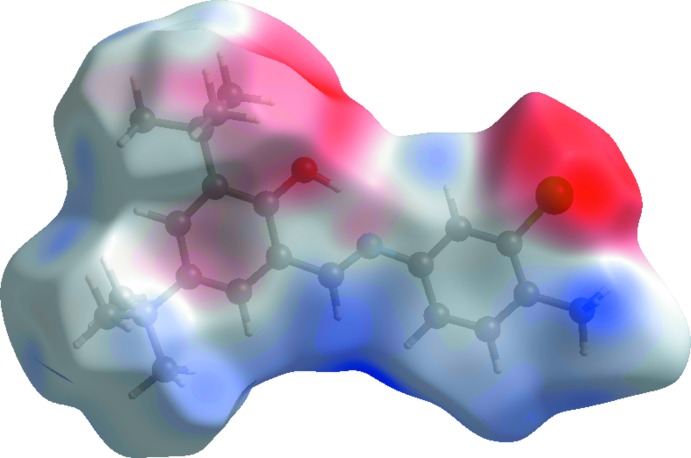
A view of the three-dimensional Hirshfeld surface of the title compound plotted over electrostatic potential energy.

A view of the three-dimensional Hirshfeld surface of the title compound plotted over electrostatic potential energy in the range −0.030 to 0.044 a.u. using the STO-3G basis set at the Hartree–Fock level of theory is shown in Fig. 7 ▸ where the C—H⋯π donors and acceptors are shown as blue and red areas around the atoms related with positive (hydrogen-bond donors) and negative (hydrogen-bond acceptors) electrostatic potentials, respectively.

## Synthesis and crystallization   

The title compound was prepared by refluxing a mixture of a solution containing 3,5-di-*tert*-butyl-2-hy­droxy­benzaldehyde (46.8 mg, 0.2 mmol) in ethanol (30 mL) and a solution containing 3-chloro-4-methyl­aniline (28.32 mg, 0.2 mmol) in ethanol (20 mL). The reaction mixture was stirred for 4 h under reflux. Crystals suitable for X-ray analysis were obtained by slow evaporation of an ethanol solution (m.p. 417–419 K; yield 84%).

## Refinement   

Crystal data, data collection and structure refinement details are summarized in Table 2 ▸. C-bound H atoms were positioned geometrically with C—H distances of 0.93–0.97 Å and refined as riding, with *U*_iso_(H) = 1.2*U*_eq_(C).

## Supplementary Material

Crystal structure: contains datablock(s) I. DOI: 10.1107/S2056989018016377/xu5948sup1.cif

Structure factors: contains datablock(s) I. DOI: 10.1107/S2056989018016377/xu5948Isup2.hkl

CCDC reference: 1873612

Additional supporting information:  crystallographic information; 3D view; checkCIF report

## Figures and Tables

**Figure 4:**
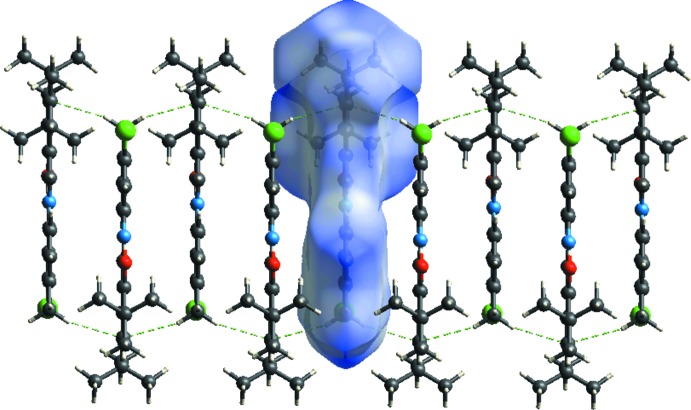
Hirshfeld surface mapped over *d*_norm_ for visualizing the inter­molecular inter­actions of the title compound.

**Table 1:** Hydrogen-bond geometry (Å, °) *Cg* is the centroid of the C9–C14 benzene ring.

*D*—H⋯*A*	*D*—H	H⋯*A*	*D*⋯*A*	*D*—H⋯*A*
O1—H1⋯N1	0.82	1.84	2.582 (3)	149
C1—H1*B*⋯*Cg*1^i^	0.96	2.77	3.5072 (4)	134

Symmetry code: (i) 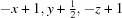.

**Table 2:** Experimental details

Crystal data	
Chemical formula	C_22_H_28_ClNO
*M*_r_	357.90
Crystal system, space group	Monoclinic, *P*2_1_/*m*
Temperature (K)	296
*a*, *b*, *c* (Å)	9.6753 (10), 7.0072 (6), 15.3749 (13)
β (°)	93.425 (7)
*V* (Å^3^)	1040.51 (17)
*Z*	2
Radiation type	Mo *K*α
μ (mm^−1^)	0.19
Crystal size (mm)	0.74 × 0.65 × 0.48

Data collection	
Diffractometer	Stoe IPDS 2
Absorption correction	Integration (*X-RED32*; Stoe & Cie, 2002 ▸)
*T*_min_, *T*_max_	0.879, 0.940
No. of measured, independent and observed [*I* > 2σ(*I*)] reflections	5895, 2300, 1298
*R*_int_	0.028
(sin θ/λ)_max_ (Å^−1^)	0.628

Refinement	
*R*[*F* ^2^ > 2σ(*F* ^2^)], *wR*(*F* ^2^), *S*	0.053, 0.168, 1.00
No. of reflections	2300
No. of parameters	145
H-atom treatment	H-atom parameters constrained
Δρ_max_, Δρ_min_ (e Å^−3^)	0.28, −0.22

Computer programs: *X-AREA* and *X-RED* (Stoe & Cie, 2002 ▸), *SHELXLXT* (Sheldrick, 2015*a* ▸), *SHELXL2017/1* (Sheldrick, 2015*b* ▸), *ORTEP-3 for Windows* and *WinGX* (Farrugia, 2012 ▸) and *PLATON* (Spek, 2009 ▸).

## supplementary crystallographic information

### Crystal data

**Table:**

C_22_H_28_ClNO	*F*(000) = 384
*M**_r_* = 357.90	*D*_x_ = 1.142 Mg m^−^^3^
Monoclinic, *P*2_1_/*m*	Mo *K*α radiation, λ = 0.71073 Å
*a* = 9.6753 (10) Å	Cell parameters from 5548 reflections
*b* = 7.0072 (6) Å	θ = 2.1–30.7°
*c* = 15.3749 (13) Å	µ = 0.19 mm^−^^1^
β = 93.425 (7)°	*T* = 296 K
*V* = 1040.51 (17) Å^3^	Prism, orange
*Z* = 2	0.74 × 0.65 × 0.48 mm

### Data collection

**Table:**

Stoe IPDS 2 diffractometer	2300 independent reflections
Radiation source: sealed X-ray tube, 12 x 0.4 mm long-fine focus	1298 reflections with *I* > 2σ(*I*)
Detector resolution: 6.67 pixels mm^-1^	*R*_int_ = 0.028
rotation method scans	θ_max_ = 26.5°, θ_min_ = 2.1°
Absorption correction: integration (X-RED32; Stoe & Cie, 2002)	*h* = −12→12
*T*_min_ = 0.879, *T*_max_ = 0.940	*k* = −8→8
5895 measured reflections	*l* = −18→19

### Refinement

**Table:**

Refinement on *F*^2^	0 restraints
Least-squares matrix: full	Hydrogen site location: mixed
*R*[*F*^2^ > 2σ(*F*^2^)] = 0.053	H-atom parameters constrained
*wR*(*F*^2^) = 0.168	*w* = 1/[σ^2^(*F*_o_^2^) + (0.0964*P*)^2^] where *P* = (*F*_o_^2^ + 2*F*_c_^2^)/3
*S* = 1.00	(Δ/σ)_max_ < 0.001
2300 reflections	Δρ_max_ = 0.28 e Å^−^^3^
145 parameters	Δρ_min_ = −0.22 e Å^−^^3^

### Special details

**Table:**

Geometry. All esds (except the esd in the dihedral angle between two l.s. planes) are estimated using the full covariance matrix. The cell esds are taken into account individually in the estimation of esds in distances, angles and torsion angles; correlations between esds in cell parameters are only used when they are defined by crystal symmetry. An approximate (isotropic) treatment of cell esds is used for estimating esds involving l.s. planes.

### Fractional atomic coordinates and isotropic or equivalent isotropic displacement parameters (Å^2^)

**Table:**

	*x*	*y*	*z*	*U*_iso_*/*U*_eq_	
C1	0.2129 (4)	0.250000	0.2502 (2)	0.0799 (9)	
H1A	0.122313	0.250000	0.272431	0.120*	
H1B	0.223846	0.361862	0.215178	0.120*	
C2	0.3181 (3)	0.250000	0.3253 (2)	0.0633 (8)	
C3	0.2795 (3)	0.250000	0.4107 (2)	0.0703 (9)	
H3	0.185649	0.250000	0.420637	0.084*	
C4	0.3723 (3)	0.250000	0.4806 (2)	0.0700 (9)	
H4	0.340566	0.250000	0.536544	0.084*	
C5	0.5133 (3)	0.250000	0.46997 (18)	0.0556 (7)	
C6	0.5560 (3)	0.250000	0.38600 (19)	0.0600 (7)	
H6	0.649961	0.250000	0.376348	0.072*	
C7	0.4596 (3)	0.250000	0.3165 (2)	0.0639 (8)	
C8	0.5942 (3)	0.250000	0.61807 (19)	0.0568 (7)	
H8	0.502444	0.250000	0.632881	0.068*	
C9	0.7006 (3)	0.250000	0.68668 (18)	0.0521 (7)	
C10	0.6637 (3)	0.250000	0.77307 (19)	0.0566 (7)	
H10	0.570247	0.250000	0.784241	0.068*	
C11	0.7595 (3)	0.250000	0.84146 (18)	0.0571 (7)	
C12	0.8995 (3)	0.250000	0.82148 (19)	0.0620 (8)	
H12	0.966318	0.250000	0.867572	0.074*	
C13	0.9445 (3)	0.250000	0.7374 (2)	0.0590 (7)	
C14	0.8423 (3)	0.250000	0.66939 (18)	0.0551 (7)	
C15	1.0991 (3)	0.250000	0.7198 (2)	0.0722 (9)	
C16	1.1330 (2)	0.0710 (4)	0.6677 (2)	0.0980 (10)	
H16A	1.076649	0.068596	0.614163	0.147*	
H16B	1.228999	0.073060	0.655013	0.147*	
H16C	1.114737	−0.040681	0.701290	0.147*	
C17	1.1899 (4)	0.250000	0.8041 (3)	0.1274 (18)	
H17A	1.285592	0.250000	0.790824	0.191*	
H17C	1.170713	0.138138	0.837395	0.191*	
C18	0.7224 (3)	0.250000	0.9368 (2)	0.0753 (9)	
C19	0.7730 (5)	0.4303 (8)	0.9793 (2)	0.176 (2)	
H19A	0.734855	0.537623	0.947277	0.264*	
H19B	0.744536	0.434730	1.037976	0.264*	
H19C	0.872234	0.434730	0.979833	0.264*	
C20	0.5646 (4)	0.250000	0.9437 (3)	0.1131 (15)	
H20A	0.525969	0.136290	0.917256	0.170*	
H20B	0.543785	0.250000	1.003968	0.170*	
Cl1	0.52181 (11)	0.250000	0.21237 (6)	0.1032 (4)	
N1	0.6191 (2)	0.250000	0.53735 (15)	0.0599 (6)	
O1	0.8791 (2)	0.250000	0.58613 (13)	0.0748 (6)	
H1	0.809294	0.250000	0.553116	0.112*	

### Atomic displacement parameters (Å^2^)

**Table:**

	*U*^11^	*U*^22^	*U*^33^	*U*^12^	*U*^13^	*U*^23^
C1	0.088 (2)	0.066 (2)	0.083 (2)	0.000	−0.0157 (18)	0.000
C2	0.0734 (18)	0.0410 (17)	0.0749 (19)	0.000	−0.0011 (15)	0.000
C3	0.0559 (16)	0.077 (2)	0.077 (2)	0.000	0.0003 (16)	0.000
C4	0.0621 (17)	0.086 (2)	0.0633 (18)	0.000	0.0113 (15)	0.000
C5	0.0611 (16)	0.0480 (17)	0.0576 (16)	0.000	0.0032 (13)	0.000
C6	0.0630 (16)	0.0543 (18)	0.0631 (17)	0.000	0.0085 (14)	0.000
C7	0.0786 (19)	0.0547 (19)	0.0587 (16)	0.000	0.0068 (15)	0.000
C8	0.0531 (14)	0.0530 (18)	0.0649 (17)	0.000	0.0079 (13)	0.000
C9	0.0527 (14)	0.0458 (16)	0.0582 (15)	0.000	0.0074 (13)	0.000
C10	0.0546 (15)	0.0551 (18)	0.0616 (16)	0.000	0.0165 (14)	0.000
C11	0.0642 (16)	0.0530 (18)	0.0550 (15)	0.000	0.0112 (14)	0.000
C12	0.0595 (16)	0.065 (2)	0.0611 (17)	0.000	0.0016 (13)	0.000
C13	0.0554 (15)	0.0556 (18)	0.0669 (17)	0.000	0.0113 (14)	0.000
C14	0.0574 (15)	0.0524 (17)	0.0566 (16)	0.000	0.0128 (13)	0.000
C15	0.0519 (16)	0.082 (2)	0.083 (2)	0.000	0.0124 (15)	0.000
C16	0.0684 (14)	0.090 (2)	0.139 (2)	0.0156 (13)	0.0353 (16)	−0.0004 (17)
C17	0.0520 (18)	0.219 (6)	0.110 (3)	0.000	−0.002 (2)	0.000
C18	0.079 (2)	0.091 (3)	0.0563 (17)	0.000	0.0149 (16)	0.000
C19	0.204 (4)	0.235 (5)	0.095 (2)	−0.106 (4)	0.061 (3)	−0.087 (3)
C20	0.102 (3)	0.168 (4)	0.073 (2)	0.000	0.036 (2)	0.000
Cl1	0.1117 (8)	0.1390 (10)	0.0595 (5)	0.000	0.0104 (5)	0.000
N1	0.0608 (13)	0.0617 (16)	0.0578 (14)	0.000	0.0075 (11)	0.000
O1	0.0613 (11)	0.1072 (18)	0.0574 (12)	0.000	0.0166 (10)	0.000

### Geometric parameters (Å, º)

**Table:**

C1—C2	1.494 (5)	C12—C13	1.389 (4)
C1—H1A	0.9600	C12—H12	0.9300
C1—H1B	0.9600	C13—C14	1.395 (4)
C1—H1B^i^	0.9600	C13—C15	1.536 (4)
C2—C7	1.383 (4)	C14—O1	1.349 (3)
C2—C3	1.387 (4)	C15—C17	1.523 (5)
C3—C4	1.359 (4)	C15—C16	1.534 (3)
C3—H3	0.9300	C15—C16^i^	1.534 (3)
C4—C5	1.383 (4)	C16—H16A	0.9600
C4—H4	0.9300	C16—H16B	0.9600
C5—C6	1.379 (4)	C16—H16C	0.9600
C5—N1	1.412 (4)	C17—H17A	0.9600
C6—C7	1.376 (4)	C17—H17C	0.9600
C6—H6	0.9300	C17—H17C^i^	0.9600
C7—Cl1	1.744 (3)	C18—C19^i^	1.491 (4)
C8—N1	1.278 (4)	C18—C19	1.491 (4)
C8—C9	1.429 (4)	C18—C20	1.537 (5)
C8—H8	0.9300	C19—H19A	0.9600
C9—C10	1.396 (4)	C19—H19B	0.9600
C9—C14	1.412 (3)	C19—H19C	0.9600
C10—C11	1.360 (4)	C20—H20A	0.9600
C10—H10	0.9300	C20—H20B	0.9595
C11—C12	1.407 (4)	C20—H20A^i^	0.9600
C11—C18	1.530 (4)	O1—H1	0.8200
			
C2—C1—H1A	108.6	O1—C14—C13	119.7 (2)
C2—C1—H1B	109.9	O1—C14—C9	119.5 (3)
H1A—C1—H1B	109.5	C13—C14—C9	120.8 (2)
C2—C1—H1B^i^	109.91 (9)	C17—C15—C16	108.31 (19)
H1A—C1—H1B^i^	109.5	C17—C15—C16^i^	108.3 (2)
H1B—C1—H1B^i^	109.5	C16—C15—C16^i^	109.7 (3)
C7—C2—C3	114.6 (3)	C17—C15—C13	111.6 (3)
C7—C2—C1	123.8 (3)	C16—C15—C13	109.45 (18)
C3—C2—C1	121.5 (3)	C16^i^—C15—C13	109.45 (18)
C4—C3—C2	123.1 (3)	C15—C16—H16A	109.5
C4—C3—H3	118.4	C15—C16—H16B	109.5
C2—C3—H3	118.4	H16A—C16—H16B	109.5
C3—C4—C5	121.1 (3)	C15—C16—H16C	109.5
C3—C4—H4	119.5	H16A—C16—H16C	109.5
C5—C4—H4	119.5	H16B—C16—H16C	109.5
C6—C5—C4	117.6 (3)	C15—C17—H17A	109.4
C6—C5—N1	116.2 (2)	C15—C17—H17C	109.5
C4—C5—N1	126.1 (2)	H17A—C17—H17C	109.5
C7—C6—C5	120.0 (3)	C15—C17—H17C^i^	109.48 (8)
C7—C6—H6	120.0	H17A—C17—H17C^i^	109.5
C5—C6—H6	120.0	H17C—C17—H17C^i^	109.5
C6—C7—C2	123.6 (3)	C19^i^—C18—C19	115.8 (5)
C6—C7—Cl1	117.2 (2)	C19^i^—C18—C11	109.23 (18)
C2—C7—Cl1	119.2 (3)	C19—C18—C11	109.23 (18)
N1—C8—C9	123.2 (2)	C19^i^—C18—C20	105.8 (2)
N1—C8—H8	118.4	C19—C18—C20	105.8 (2)
C9—C8—H8	118.4	C11—C18—C20	110.9 (3)
C10—C9—C14	119.1 (3)	C18—C19—H19A	109.5
C10—C9—C8	119.2 (2)	C18—C19—H19B	109.5
C14—C9—C8	121.7 (2)	H19A—C19—H19B	109.5
C11—C10—C9	122.3 (2)	C18—C19—H19C	109.5
C11—C10—H10	118.9	H19A—C19—H19C	109.5
C9—C10—H10	118.9	H19B—C19—H19C	109.5
C10—C11—C12	116.9 (2)	C18—C20—H20A	109.5
C10—C11—C18	123.5 (2)	C18—C20—H20B	109.4
C12—C11—C18	119.6 (3)	H20A—C20—H20B	108.1
C13—C12—C11	124.2 (3)	C18—C20—H20A^i^	109.49 (9)
C13—C12—H12	117.9	H20A—C20—H20A^i^	112.2
C11—C12—H12	117.9	H20B—C20—H20A^i^	108.1
C12—C13—C14	116.8 (2)	C8—N1—C5	122.8 (2)
C12—C13—C15	121.8 (3)	C14—O1—H1	109.5
C14—C13—C15	121.5 (3)		
			
C7—C2—C3—C4	0.000 (1)	C12—C13—C14—O1	180.000 (1)
C1—C2—C3—C4	180.000 (1)	C15—C13—C14—O1	0.000 (1)
C2—C3—C4—C5	0.000 (1)	C12—C13—C14—C9	0.000 (1)
C3—C4—C5—C6	0.000 (1)	C15—C13—C14—C9	180.000 (1)
C3—C4—C5—N1	180.000 (1)	C10—C9—C14—O1	180.000 (1)
C4—C5—C6—C7	0.000 (1)	C8—C9—C14—O1	0.000 (1)
N1—C5—C6—C7	180.000 (1)	C10—C9—C14—C13	0.000 (1)
C5—C6—C7—C2	0.000 (1)	C8—C9—C14—C13	180.000 (1)
C5—C6—C7—Cl1	180.000 (1)	C12—C13—C15—C17	0.000 (1)
C3—C2—C7—C6	0.000 (1)	C14—C13—C15—C17	180.000 (1)
C1—C2—C7—C6	180.000 (1)	C12—C13—C15—C16	119.9 (2)
C3—C2—C7—Cl1	180.000 (1)	C14—C13—C15—C16	−60.1 (2)
C1—C2—C7—Cl1	0.000 (1)	C12—C13—C15—C16^i^	−119.9 (2)
N1—C8—C9—C10	180.000 (1)	C14—C13—C15—C16^i^	60.1 (2)
N1—C8—C9—C14	0.000 (1)	C10—C11—C18—C19^i^	116.2 (3)
C14—C9—C10—C11	0.000 (1)	C12—C11—C18—C19^i^	−63.8 (3)
C8—C9—C10—C11	180.000 (1)	C10—C11—C18—C19	−116.2 (3)
C9—C10—C11—C12	0.000 (1)	C12—C11—C18—C19	63.8 (3)
C9—C10—C11—C18	180.000 (1)	C10—C11—C18—C20	0.000 (2)
C10—C11—C12—C13	0.000 (2)	C12—C11—C18—C20	180.000 (1)
C18—C11—C12—C13	180.000 (1)	C9—C8—N1—C5	180.000 (1)
C11—C12—C13—C14	0.000 (1)	C6—C5—N1—C8	180.000 (1)
C11—C12—C13—C15	180.000 (1)	C4—C5—N1—C8	0.000 (1)

Symmetry code: (i) *x*, −*y*+1/2, *z*.

### Hydrogen-bond geometry (Å, º)

Cg is the centroid of the C9–C14 benzene ring.

**Table:**

*D*—H···*A*	*D*—H	H···*A*	*D*···*A*	*D*—H···*A*
O1—H1···N1	0.82	1.84	2.582 (3)	149
C1—H1*B*···*Cg*1^ii^	0.96	2.77	3.5072 (4)	134

Symmetry code: (ii) −*x*+1, *y*+1/2, −*z*+1.

## References

[bb1] Atalay, Ş., Gerçeker, S., Meral, S. & Bülbül, H. (2017). *IUCrData*, **2**, x171725.

[bb2] Calligaris, M., Nardin, G. M. J. & Randaccio, C. (1972). *Coord. Chem. Rev.* **7**, 385–403.

[bb3] Elmalı, A., Kabak, M., Kavlakoğlu, E. & Elerman, Y. (1999). *J. Mol. Struct.* **510**, 207–214.

[bb4] El-masry, A. H., Fahmy, H. H. & Ali Abdelwahed, S. (2000). *Molecules*, **5**, 1429–1438.

[bb5] Farrugia, L. J. (2012). *J. Appl. Cryst.* **45**, 849–854.

[bb6] Groom, C. R., Bruno, I. J., Lightfoot, M. P. & Ward, S. C. (2016). *Acta Cryst.* B**72**, 171–179. 10.1107/S2052520616003954 PMC4822653 27048719

[bb7] Gumus, M. K., Kansiz, S., Dege, N. & Kalibabchuk, V. A. (2018). *Acta Cryst.* E**74**, 1211–1214. 10.1107/S2056989018010848 PMC6127710 30225101

[bb8] Hökelek, T., Bilge, S., Demiriz, Ş., Özgüç, B. & Kılıç, Z. (2004). *Acta Cryst.* C**60**, o803–o805. 10.1107/S0108270104022565 15528825

[bb9] Iwan, A., Kaczmarczyk, B., Janeczek, H., Sek, D. & Ostrowski, S. (2007). *Spectrochim. Acta A Mol. Biomol. Spectrosc.* **66**, 1030–1041. 10.1016/j.saa.2006.05.016 16872877

[bb10] Kansız, S. & Dege, N. (2018). *J. Mol. Struct.* **1173**, 42–51.

[bb11] Kansiz, S., Macit, M., Dege, N. & Tsapyuk, G. G. (2018). *Acta Cryst.* E**74**, 1513–1516. 10.1107/S2056989018013117 PMC6176450 30319813

[bb12] Moroz, Y. S., Demeshko, S., Haukka, M., Mokhir, A., Mitra, U., Stocker, M., Müller, P., Meyer, F. & Fritsky, I. O. (2012). *Inorg. Chem.* **51**, 7445–7447. 10.1021/ic300902z 22765646

[bb13] Sen, P., Kansiz, S., Golenya, I. A. & Dege, N. (2018). *Acta Cryst.* E**74**, 1147–1150. 10.1107/S2056989018009726 PMC6073007 30116581

[bb14] Şen, F., Kansiz, S. & Uçar, İ. (2017). *Acta Cryst.* C**73**, 517–524. 10.1107/S2053229617008452 28677602

[bb15] Sheldrick, G. M. (2015*a*). *Acta Cryst.* A**71**, 3–8.

[bb16] Sheldrick, G. M. (2015*b*). *Acta Cryst.* C**71**, 3–8.

[bb17] Spek, A. L. (2009). *Acta Cryst.* D**65**, 148–155. 10.1107/S090744490804362X PMC2631630 19171970

[bb18] Stoe & Cie (2002). *X-AREA* and *X-RED32*. Stoe & Cie GmbH, Darmstadt, Germany.

[bb19] Su, D.-C., Wang, F.-T., Mao, C.-G. & Qian, S.-S. (2012). *Acta Cryst.* E**68**, o303. 10.1107/S1600536811055590 PMC3274996 22346941

[bb20] Toprak, Ş., Tanak, H., Macit, M., Dege, N. & Orbay, M. (2018). *J. Mol. Struct.* **1174**, 184–191.

[bb21] Turner, M. J., MacKinnon, J. J., Wolff, S. K., Grimwood, D. J., Spackman, P. R., Jayatilaka, D. & Spackman, M. A. (2017). *CrystalExplorer17.5*. University of Western Australia, Perth.

[bb22] Wang, H. (2010). *Acta Cryst.* E**66**, m1571. 10.1107/S1600536810046088 PMC3011529 21589258

